# The Impact of Kidney Transplantation on the Serum Fatty Acid Profile in Patients with End-Stage Kidney Disease

**DOI:** 10.3390/nu14040772

**Published:** 2022-02-12

**Authors:** Maciej Śledziński, Aleksandra Hliwa, Justyna Gołębiewska, Adriana Mika

**Affiliations:** 1 Department of General, Endocrine and Transplant Surgery, Faculty of Medicine, Medical University of Gdansk, 80-214 Gdansk, Poland; msledz@gumed.edu.pl; 2 Department of Pharmaceutical Biochemistry, Faculty of Pharmacy, Medical University of Gdansk, 80-211 Gdansk, Poland; aleksandra.hliwa@gumed.edu.pl; 3 Department of Nephrology, Transplantology and Internal Medicine, Medical University of Gdansk, 80-211 Gdansk, Poland; jgolebiewska@gumed.edu.pl

**Keywords:** fatty acids, kidney transplant, end-stage kidney disease, nutrition

## Abstract

Epidemiological data indicate that metabolic disturbances and increased cardiovascular risk in renal transplant patients are a significant and common problem. Therefore, it is important to search for new solutions and, at the same time, counteract the negative effects of currently used therapies. In this study, we examined the effect of kidney transplantation on the serum levels of fatty acids (FAs) in order to assess the role of these compounds in the health of transplant patients. The FA profile was analyzed by gas chromatography-mass spectrometry in the serum of 35 kidney transplant recipients, just before transplantation and 3 months later. The content of total n-3 polyunsaturated FAs (PUFAs) decreased after transplantation (3.06 ± 0.13% vs. 2.66 ± 0.14%; *p* < 0.05). The total amount of ultra-long-chain FAs containing 26 and more carbon atoms was significantly reduced (0.08 ± 0.009% vs. 0.05 ± 0.007%; *p* < 0.05). The desaturation index (18:1/18:0) increased after transplantation (3.92 ± 0.11% vs. 4.36 ± 0.18%; *p* < 0.05). The study showed a significant reduction in n-3 PUFAs in renal transplant recipients 3 months after transplantation, which may contribute to increased cardiovascular risk in this patient population.

## 1. Introduction

Despite advances in renal replacement therapy, the mortality rate in patients with chronic kidney disease (CKD) remains several times higher than in the general population. CKD is associated with an increased risk of cardiovascular disease (CVD) [1]. Dyslipidemia associated with CKD, which includes elevated triglycerides (TG), low-density lipoprotein cholesterol (LDL), total cholesterol (CHOL), and a low concentration of high-density lipoprotein cholesterol (HDL) [1], may promote the process of atherosclerosis and contribute to a high rate of cardiovascular complications [2].

End-stage CKD requires renal replacement therapy (RRT), including dialysis, which keeps patients alive, yet at the same time, exposes them to many complications, including CVD. Kidney transplantation (KTx) either from a living donor (most often a family member) or from a deceased donor seems to be the RRT of choice for the majority of patients. The quality of life and life expectancy significantly improve after kidney transplantation; therefore, it is the optimal method of CKD treatment despite the fact that it is associated with the increased risk of obesity, post-transplant diabetes, arterial hypertension, and other adverse metabolic effects of immunosuppressive treatment. The risk of CVD decreases after KTx but still remains a major cause of morbidity and mortality among these patients [3].

A major fraction of lipids, fatty acids (FAs) are a structural component of most complex lipids or can circulate in the blood in a free form (bound with albumin). FAs build the phospho- and glycolipids of biological membranes and create fatty energy stores in the form of TG [4]. The metabolic balance of the body can be disturbed by changes in the blood profile of FAs and their absolute number. Disorders in the FA profile are associated with oxidative stress, inflammation, lipotoxicity, and hypertriglyceridemia and might predispose to cardiovascular risk [5].

CKD promotes FA disorders by metabolic alterations and dietary restrictions resulting from chronic intoxication. Previous research has shown changes in the proportion of individual serum FAs in CKD patients; in particular, the content of serum monounsaturated FAs (MUFAs) is increased [6], and the content of n-3 polyunsaturated FAs (PUFAs) is decreased [7,8,9,10,11].

Most studies indicate that n-3 PUFAs may contribute to cardiovascular protection in the general population [12,13]. N-3 PUFAs reduce oxidative stress and exhibit anti-inflammatory properties by reducing the production of pro-inflammatory cytokines and the expression of adhesion molecules [14]. They are also believed to have anti-aggregating and anti-atherosclerotic effects. Our team’s research showed a significantly lower PUFA content in dialysis patients, which may contribute to their high cardiovascular risk [8]. That study also showed that patients with a higher content of adipose tissue had a favorable composition of FAs [8].

The dominant group of FAs are even-chain FAs [15]. A smaller part are FAs with odd chains (OCFAs) and branched-chain FAs (BCFAs). Many FAs affect the metabolism of cells and the whole organism; hence, they can be classified as bioactive agents. This group includes n-3 PUFAs, BCFAs, and OCFAs, which have some beneficial health effects [16].

Kidney transplantation, like any therapy, has negative consequences. Basic lipid disorders are known to occur both in patients with end-stage kidney disease and in kidney transplant recipients.

Only a few publications in the literature investigate the issue of the FA profile in kidney transplant recipients. Most of them assess the effect of n-3 PUFA supplementation on the kidney allograft function and lipid profile [17,18].

In our previous studies, we observed that despite normal kidney function after transplantation, the FA profile was still abnormal [19]. Our recent work concerning a comparison of FA profiles in patients at different time intervals after transplantation and healthy controls showed many differences in FA profiles [20]. In that study, however, we were unable to evaluate the same patients before and after transplantation, as we did in this study. Therefore, the aim of this study was to investigate the real effect of the kidney transplant procedure on the FA profile. To the best of our knowledge, this is the first such study to compare the serum FA profile of the same patients before and after transplantation.

## 2. Materials and Methods

The study included 35 CKD patients (19 men and 16 women) aged 22–70 years, evaluated before kidney transplantation from deceased donor and 3 months after the transplantation procedure. Unfortunately, we were not able to include in this study patients who had kidney transplantation from living donors due to the insufficient number of these transplantations in our center. In order to investigate the effect of transplantation on the FA profile, serum samples were collected at 2 time points from the same patients. The samples were collected just before transplantation and 3 months after the transplantation. The kidney transplant process begins when the deceased donor is qualified for organ donation. After tissue typing is performed, the recipient is selected from a nationwide list. The selected recipient is transported to the center that performs the procurement. Only recipients from the Pomeranian Region were included in the study, which allowed for further follow-up observations. The consent of the Independent Bioethics Committee of the Medical University of Gdansk was obtained (protocol no. NKBBN 151212019 of 4 September 2019) to perform this study. Written informed consent was obtained after the patient was admitted on the day of the transplant. After preliminary preparation and fasting, a blood sample was taken for analysis. After centrifugation at 3000× *g*, the serum sample was coded and stored at −80 °C until the FA analysis.

After a successful transplantation, the patients underwent further follow-up at the Nephrology Outpatient Clinic. Patients received dietary instructions in accordance with Polish guidelines [21]. Three months after the kidney transplantation, a fasting blood sample was taken again for analysis as part of a routine check-up. Serum was secured in the same way as on the day of transplantation. All but one patient were treated with three immunosuppressants—cyclosporine or tacrolimus and mycophenolate mofetil and steroids. Half of this group of patients were additionally induced with basiliximab or thymoglobulin (Table 1). Patients receiving FA supplementation, patients with abnormal kidney transplant function during the follow-up period, patients who did not consent to the study, and those who were not present for the follow-up were excluded from the study. A participation success rate of 81% was achieved.

The FA profile was analyzed by gas chromatography with mass spectrometry (GC-MS), using the same method as in the previous work [20]. Briefly, total lipids were extracted from each patient’s serum with a mixture of chloroform-methanol (2:1, *v*/*v*), according to the Folch et al. method [22]. Next, a total lipid extract from each sample was subjected to 3 h of hydrolysis with 0.5 M KOH at 90 °C. After that, the mixture was neutralized with 6 M HCl. Next, 1 mL of water was added, and unesterified FAs were extracted three times with 1 mL of *n*-hexane. The organic phase was evaporated under a nitrogen stream. Extracts were then derivatized into FA methyl esters (FAMEs) with 10% BF_3_ in methanol solution for 1.5 h at 55 °C. Then, 1 mL of water was added, and FAMEs were extracted with 3 × 1 mL of *n*-hexane, dried under a nitrogen stream, and stored at −20 °C until the GC-MS analysis. The FAMEs were detected by a GC-EI-MS QP-2010SE spectrometer (Shimadzu, Kyoto, Japan) with chromatographic separation on a Zebron ZB-5MSi capillary column, 30 m × 0.25 mm i.d. × 0.25-µm film thickness (Phenomenex, Torrance, CA, USA). Separation parameters were set as follows: column oven temperature was 60–300 °C (4 °C/min), total analysis run time was 60 min; helium was used as a carrier gas (column head pressure at 100 kPa). The MS analysis was conducted in full scan mode, with the mass scan range set at *m*/*z* 45–700. The electron impact source operated at 70 eV. FAs were identified using a reference standards mixture (37 FAME Mix, Sigma Aldrich, St. Louis, MO, USA) and the reference library NIST 11. The internal standard was 19-methylarachidic acid.

### Statistical Analysis

In the analysis, the significance of changes was assessed using the two-tailed Student’s *t*-test for parametric data. For nonparametric data, the Mann–Whitney U test was used. Normal distribution was assessed with the Shapiro–Wilk test. We assumed the significance of the differences at the level of *p* < 0.05. The results were presented as means together with the standard error of the mean (SEM). The Sigma Plot 14.5 program (Systat Software, San Jose, CA, USA) was used for the calculations.

## 3. Results

As expected, patients 3 months after kidney transplantation had significant improvements in renal function parameters (GFR, creatinine, BUN). The C-reactive protein (CRP), which serves as an indicator of inflammation, was also significantly reduced (Table 1).

**Figure 1:**
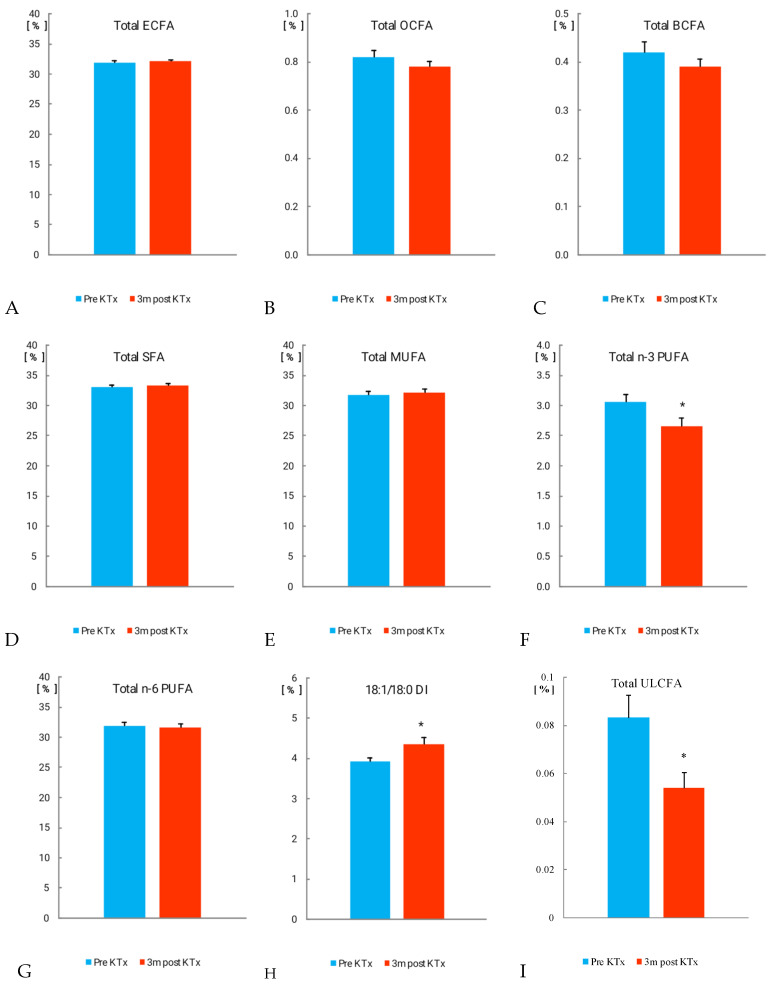
Fatty acid content (%) in patient serum. Values are mean ± SEM. * *p* < 0.05. (**A**) Total ECFA, even-chain fatty acids; (**B**) Total OCFA, odd-chain fatty acids; (**C**) Total BCFA, branched-chain fatty acids; (**D**) Total SFA, saturated fatty acids; (**E**) Total MUFA, monounsaturated fatty acids; (**F**) Total n-3 PUFA, n-3 polyunsaturated fatty acids; (**G**) Total n-6 PUFA, n-6 polyunsaturated fatty acids; (**H**) 18:1/18:0 DI, desaturation index; (**I**) Total ULCFA, ultra-long-chain fatty acid (26:0–32:0).

The total even-chain saturated FAs (ECFAs), OCFAs, BCFAs, and total SFAs did not change after transplantation (Figure 1A–D). However, among ECFAs, the amounts of FAs containing 12, 18, and 26 carbon atoms were significantly lower 3 months after transplantation than before the procedure (Supplementary Table S1). The opposite was observed in the case of palmitic acid (16:0), the amount of which increased.

**Figure 2:**
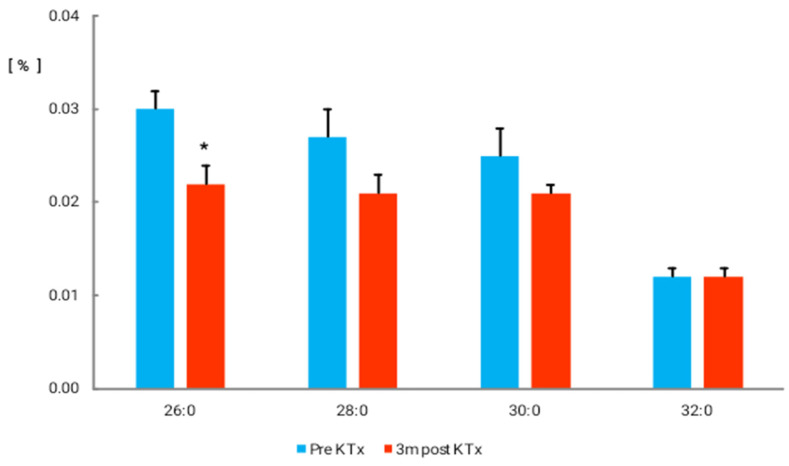
Ultra-long-chain fatty acid (ULCFA) content (%) in patient serum. Values are mean ± SEM. * *p*< 0.05.

The total amount of ultra-long-chain FAs (ULCFAs) containing 26 and more carbon atoms was significantly reduced (Figure 1I; 0.08 ± 0.009% vs. 0.05 ± 0.007%; *p* < 0.05). Among them, 26:0 was significantly decreased 3 months after kidney transplantation (0.03 ± 0.002% vs. 0.022 ± 0.002%; *p* < 0.05), whereas 28:0 and 30:0 tended to decrease (Figure 2).

Among monounsaturated fatty acids (MUFAs), after transplantation, a statistically significant increase was found only for 20:1 (Supplementary Table S1), but the total MUFAs did not change (Figure 1E). We also found an increase in 18:1/18:0 desaturation index (DI; Figure 1H; 3.92 ± 0.11% vs. 4.36 ± 0.18%; *p* < 0.05).

The proportion of some n-6 PUFAs was significantly decreased 3 months after KTx, as was adrenic acid (22:4n-6; AdA), dihomo-γ-linolenic acid (20:3n-6; DGLA), and arachidonic acid (ARA), whereas 18:2n-6 increased after transplantation (Table 2).

The total n-6 PUFAs were not changed 3 months after kidney transplantation (Figure 1G). The content of total n-3 PUFAs decreased after transplantation (Figure 1F; 3.06 ± 0.13% vs. 2.66 ± 0.14%; *p* <0.05), and most of them decreased statistically significantly (Table 2).

## 4. Discussion

Metabolic disorders, including impairment of the FA profile, are among a variety of symptoms typical for CKD [23]. Kidney transplantation is by far the most optimal method of renal replacement therapy, leading to the resolution of many disorders in these patients. Our previous work [20] showed an altered FA profile in patients with kidney transplant compared to healthy controls. The effect of kidney transplantation on changes in the FA profile is not well studied. Many FAs play a very important role in various processes in humans (e.g., inflammation, insulin resistance, energy metabolism, oxidative stress); thus, it is crucial to assess the exact impact of the treatment on their profile [24].

In the present study, among the groups of FAs, we found a significant change after transplantation in the case of total n-3 PUFAs. The observed reduction in n-3 PUFAs may have a negative impact on the health of patients after kidney transplantation. Compounds that exhibit anti-inflammatory and immunomodulatory properties are formed from this group of FAs [25]. The main substrates for the synthesis of these anti-inflammatory compounds are DHA and EPA. The remaining n-3 PUFAs, ETA and DPA, are precursors in the synthesis of DHA from essential FA–ALA. In turn, ALA and ETA are substrates in the synthesis of EPA [26]. It is possible that the decrease in the amount of n-3 PUFAs and some n-6 PUFAs (ARA, DGLA) is related to the increased production of oxylipins. Oxylipins are biologically active lipid compounds that are formed from PUFAs. Oxylipins originating from n-3 PUFAs most often have an anti-inflammatory effect. In contrast, those arising from n-6 PUFAs can promote the inflammation process [27]; however, some CYP-mediated metabolites of ARA (n-6 PUFA), which are epoxyeicosatrienoic acids, have anti-inflammatory and cardioprotective properties [28]. Furthermore, AdA may play a role in resolving inflammation by blocking LBT4 production by neutrophils [29]. After kidney transplantation, anti-inflammatory mechanisms predominate in the body, which was confirmed here by the CRP measurement (Table 1). Inflammation reduction is certainly a complex process involving the effects of immunosuppressants and the absence of pro-inflammatory factors associated with chronic renal failure. Experimental studies and studies conducted on the general population have shown various effects of n-3 PUFAs, including the reduction of cardiovascular risk, the decreased production of triglycerides in the liver, lower heart rate, increased cardiac efficiency, decreased blood pressure and vasodilatation, and reduced blood clotting [30]. The supplementation of n-3 PUFAs might contribute to the reduction of cardiovascular risk; however, there are currently no such recommendations for kidney transplant recipients [31]. That is why research in this group of patients is of crucial importance. Our results suggest that n-3 PUFA supplementation might be worth considering in these patients.

Stearoyl-CoA desaturase 1 (SCD1) plays an important role in the synthesis of unsaturated acids. In the liver and adipose tissue, this enzyme catalyzes the process of biosynthesis of MUFAs, the major components of TG [32]. The activity of this enzyme can be determined indirectly using the DI [33]. The DI is the ratio of the desaturation product (MUFA—18:1) to the substrate (SFA—18:0) for the assessment of SCD1 activity. Three months after transplantation, we found a significant increase in the DI, which indicates an increase in the activity of SCD1 in the liver and/or in adipose tissue. This observation, in the perspective of time, may mean a negative phenomenon because it is associated with an increase in serum MUFAs and TG [34]. However, in our patients, neither MUFAs nor TG changed significantly after kidney transplantation, and the increase in the DI was rather caused by the decrease in the substrate 18:0.

An interesting finding was a decrease in ULCFAs with 26 and longer carbon chains, which may be related to their increased degradation that occurs in the liver [35]. We observed the opposite situation in our latest study (not yet published) assessing the FA profile during liver transplantation. We found a significant increase in the levels of very long-chain fatty acids (VLCFAs, with 20 or more carbon atoms in their chains) after completion of the anhepatic phase of transplantation, indicating the importance of VLCFA degradation in the liver peroxisomes. Thus, the decrease in ULCFAs after kidney transplantation may suggest the improvement of liver function in our patients. The decrease in ULCFAs after kidney transplantation may be a favorable phenomenon, considering that an increase in VLCFAs has been shown to be associated with a higher risk of CVD [36,37]. Nevertheless, in the longer term after transplantation, an increase in ULCFAs may be expected because it has been shown that a large proportion of kidney transplant patients develop liver dysfunction, and the most common known cause is the hepatotoxicity of immunosuppressants [38,39].

The current study showed an alteration of patients’ FA profiles due to transplantation by comparing the serum FA profile in patients pre- and post- transplantation. This profile can be influenced by many factors. The fact that these patients were immunosuppressed may have had some impact. Similarly, other medications that patients were taking may have altered the lipid metabolism. Together with immunosuppression, each recipient took an average of 11 types of drugs after discharge from the hospital. We could not in any way eliminate or evaluate the influence of these substances on the examined FA profiles. The size of the study group and the large number of medications taken did not allow us to divide patients into groups depending on the type of medications taken. It should also be added that renal failure is usually associated with the presence of CVD and a multitude of complications of chronic toxemia in the course of CKD and hemodialysis [40]. Diet is an important element influencing the FA profile. Patients with renal insufficiency usually follow a balanced and easily digestible diet for many years. Patients with CKD before and after transplantation have similar dietary recommendations [21], an important element of which is to consume products with a low glycemic index. They are also recommended to maintain a healthy lifestyle and physical activity. The fact of kidney transplantation in a short period of several months does not affect dietary habits; thus, we can assume that this element did not interfere with the results of the study.

Our previous study [20] showed significantly greater deviations in the FA profile in patients after transplantation, which may be due to the fact that it was a study comparing transplant patients with healthy controls. The many metabolic changes [41] and slow recovery post-surgery in patients with CKD may also contribute to the deviation in the FA profile observed in our previous study. Therefore, the results of the current study are expectedly different from the previous study [20]. Even though the duration of the current study was relatively short, the alteration of the FA profile was significant.

After transplantation, recipients are particularly vulnerable to complications related to intensive immunosuppressive therapy, such as wound-healing disorders, infections, peptic ulcer disease, pancreatitis, and stroke. However, cardiovascular complications are the most common and the most important. Only over a long period of time are the greater benefits noticeable, i.e., the reduction of complications related to chronic dialysis and the prolongation of life [42].

It is known that disturbances in the FA profile increase cardiovascular risk. Our research allowed us to observe exactly what changes occurred in the FA profile after kidney transplantation. The results suggest that decreased n-3 PUFAs may increase the cardiovascular risk in this group of patients. Moreover, the obtained results may suggest that the supplementation of appropriate FAs, especially n-3 PUFAs, alone or with dietary intervention, may reduce disorders in the FA profile and cardiovascular risk, but this should be confirmed in a properly designed clinical trial.

Among the limitations of this study is the relatively small number of patients, which did not allow to perform a multivariable analysis to adjust the changes in various FA for potentially confounding variables, such as CHOL and TG. However, due to applying paired statistical tests, we were able to obtain conclusive results in this group of CKD patients.

## 5. Conclusions

This study showed that kidney transplantation in patients suffering from CKD changes their FA profile over a 3-month follow-up period, and the most clear-cut changes concern a significant decrease in n-3 PUFAs. These results confirm our previous studies comparing transplant patients with healthy people, which also showed decreased levels of n-3 PUFAs in patients after kidney transplantation. Since such disorders may increase cardiovascular risk, it seems that further research with the use of nutritional intervention for supplementing the deficiencies shown is necessary.

## Figures and Tables

**Table 1:** Results of biochemical tests, clinical characteristics, and the anthropometric evaluation of patients. Values are mean ± SEM.

	Pre KTx	3 m Post KTx	*p*	Reference Value
	*n* = 35	*n* = 35		
Age	49.9 ± 2.41			
Sex	M: 19 F: 16			
BMI (kg/m^2^)	26.1 ± 0.56	26.0 ± 0.53	NS	18.5–24.9
laboratory tests				
Hemoglobin (g/dL)	11.0 ± 0.24	12.3 ± 0.32	<0.05	13.0–17.0
eGFR-CKD (mL/min/1.73 m^2^)	8.06 ± 0.53	49.0 ± 2.38	<0.05	>60
BUN (mg/dL)	43.8 ± 1.98	28.0 ± 1.98	<0.05	8.9–20.6
CRP (mg/L)	8.99 ± 1.80	2.60 ± 0.96	<0.05	0.0–5.0
Glucose (mg/dL)	106 ± 7.13	112 ± 6.26	NS	70–99
Creatinine (mg/dL)	7.59 ± 0.45	1.54 ± 0.07	<0.05	0.7–1.3
Albumin (g/L)	35.3 ± 0.97	37.7 ± 1.42	NS	35–50
Sodium (mmol/L)	140 ± 0.47	140 ± 0.39	NS	136–145
Potassium (mmol/L)	4.56 ± 0.11	4.36 ± 0.07	NS	3.5–5.1
ALT (U/L)	17.7 ± 1.49	25.7 ± 2.87	NS	<55
AST (U/L)	18.4 ± 1.34	29.2 ± 8.92	NS	5–34
TG (mg/dL)	251 ± 42.1	202 ± 22.2	NS	<150
CHOL (mg/dL)	205 ± 12.3	233 ± 8.71	<0.05	115–190
immunosuppressive drugs				
MP + Tc + MM	*n* = 15			
MP + Cc + MM	*n* = 2			
MP + Tc	*n* = 1			
MP + Cc + MM + Ba	*n* = 1			
MP + Tc + MM + Ba	*n* = 8			
MP + Tc + MM + Th	*n* = 8			
renal replacement therapy before KTx				
PreEmptive	*n* = 3			
Peritoneal Dialysis	*n* = 3			
Hemodialysis	*n* = 29			
Delayed Graft Function	*n* = 6			

Pre KTx, just before kidney transplant surgery; 3 m post KTx, 3 months after kidney transplantation; ALT, Alanine aminotransferase; AST, Aspartate aminotransferase; Ba, Basiliximab; BMI, body mass index; BUN, blood urea nitrogen; Cc, Ciclosporin; CHOL, total cholesterol; CRP, C-reactive protein; eGFR-CKD, estimated glomerular filtration rate using CKD-EPI creatinine equation; HDL, high-density lipoprotein; NS, not significant; MP, Methylprednisolone; MM, Mycophenolate mofetil; Tc, Tacrolimus; Th, Thymoglobulin; TG, Triacylglycerols.

**Table 2:** Polyunsaturated fatty acid content (%) in patient serum. Values are mean ± SEM.

	PreKTx	3 m Post KTx	*p*
16:2n-6	0.011 ± 0.004	0.012 ± 0.004	0.423
18:2n-6 (LA)	24.1 ± 3.89	25.4 ± 3.46	0.020
20:2n-6	0.12 ± 0.04	0.14 ± 0.04	0.123
20:3n-6 (DGLA)	1.20 ± 0.40	0.87 ± 0.32	<0.001
20:4n-6 (ARA)	6.33 ± 1.39	5.15 ± 1.07	<0.001
22:4n-6 (AdA)	0.16 ± 0.04	0.13 ± 0.04	<0.001
18:3n-3 (ALA)	0.29 ± 0.13	0.24 ± 0.16	0.094
20:4n-3 (ETA)	0.078 ± 0.023	0.061 ± 0.030	<0.001
20:5n-3 (EPA)	0.74 ± 0.25	0.73 ± 0.43	0.916
22:5n-3 (DPA)	0.44 ± 0.10	0.38 ± 0.08	0.007
22:6n-3 (DHA)	1.51 ± 0.51	1.24 ± 0.45	0.001

Pre KTx, just before kidney transplant surgery; 3 m post KTx, 3 months after kidney transplantation; LA, linoleic acid (18:2n-6); eicosadienoic acid (20:2n-6); DGLA, dihomo-γ-linolenic acid (20:3n-6); ARA, arachidonic acid (20:4n-6); AdA, adrenic acid (22:4n-6); ALA, α-linolenic acid (18:3n-3); ETA, eicosatetraenoic acid (20:4n-3); EPA, eicosapentaenoic acid (20:5n-3); DPA, docosapentaenoic acid (22:5n-3); DHA, docosahexaenoic acid (22:6n-3).

## Data Availability

Data available on request due to restrictions eg privacy or ethical. The data presented in this study are available on request from the corresponding author. The data are not publicly available due to the need to maintain the confidentiality of research results, which was included in the patients’s consent and aprproved by the bioethics committee.

## Supplementary Materials

The following supporting information can be downloaded at: https://www.mdpi.com/article/10.3390/nu14040772/s1, Table S1: Individual fatty acid content (%) in patient serum before and 3 months after kidney transplantation. Values are mean ± SEM.
