# Supplementary material for: The Impact of Kidney Transplantation on the Serum Fatty Acid Profile in Patients with End-Stage Kidney Disease
Source: Nutrients. 2022 Feb 12;14(4):772. doi: 10.3390/nu14040772 (PMC8876092; doi:10.3390/nu14040772)
Supplement: Supplementary file 1 [file nutrients-14-00772-s001.zip › nutrients-1556814-supplementary.pdf]

nutrients

MDPI

Article

The Impact of Kidney Transplantation on the Serum Fatty Acid Profile in Patients with End-Stage Kidney Disease

Maciej Śledziński [1],Aleksandra Hliwa [2], Justyna Gołębiewska [3], and Adriana Mika [2,*]

[1] Department of General, Endocrine and Transplant Surgery, Faculty of Medicine, Medical University of Gdansk, 80-214 Gdansk, Poland; msledz@gumed.edu.pl
[2] Department of Pharmaceutical Biochemistry, Faculty of Pharmacy, Medical University of Gdansk, 80-211 Gdansk, Poland; aleksandra.hliwa@gumed.edu.pl, adriana.mika@gumed.edu.pl
[3] Department of Nephrology, Transplantology and Internal Medicine, Medical University of Gdansk, 80-211 Gdansk, Poland; jgolebiewska@gumed.edu.pl
* Correspondence: adriana.mika@gumed.edu.pl

Supplementary Table S1. Individual fatty acid content (%) in patient's serum before and 3 months after kidney transplantation. Values are mean ± SEM.

	PreKTx	3m post KTx	p
10:0	0.010 ± 0.000	0.013 ± 0.001	0.333
12:0	0.21 ± 0.02	0.16 ± 0.01	0.048
14:0	1.27 ± 0.08	1.33 ± 0.09	0.591
16:0	22.5 ± 0.27	23.3 ± 0.29	0.025
18:0	7.28 ± 0.20	6.77 ± 0.20	0.041
20:0	0.11 ± 0.01	0.12 ± 0.01	0.077
22:0	0.20 ± 0.01	0.19 ± 0.01	0.456
24:0	0.22 ± 0.01	0.20 ± 0.01	0.159
26:0	0.030 ± 0.002	0.022 ± 0.002	0.006
28:0	0.027 ± 0.003	0.021 ± 0.002	0.207
30:0	0.025 ± 0.003	0.021 ± 0.001	0.337
32:0	0.012 ± 0.001	0.012 ± 0.001	1.000
11:0	0.009 ± 0.001	0.003 ± 0.001	0.058
13:0	0.013 ± 0.001	0.013 ± 0.001	0.676
15:0	0.34 ± 0.02	0.32 ± 0.01	0.348
17:0	0.31 ± 0.01	0.29 ± 0.01	0.086
19:0	0.020 ± 0.001	0.020 ± 0.001	1.000
21:0	0.018 ± 0.002	0.016 ± 0.001	0.303
23:0	0.068 ± 0.005	0.067 ± 0.004	0.906
25:0	0.051 ± 0.005	0.050 ± 0.010	0.890
14:1	0.061 ± 0.007	0.073 ± 0.009	0.273
16:1	3.31 ± 0.15	3.16 ± 0.15	0.136

18:1	28.0 ± 0.49	28.5 ± 0.54	0.342
19:1	0.014 ± 0.001	0.015 ± 0.001	0.689
20:1	0.14 ± 0.009	0.16 ± 0.008	0.016
22:1	0.019 ± 0.002	0.020 ± 0.002	0.594
24:1	0.27 ± 0.02	0.29 ± 0.02	0.262
16:2n-6	0.011 ± 0.004	0.012 ± 0.004	0.423
18:2n-6 (LA)	24.1 ± 3.89	25.4 ± 3.46	0.020
20:2n-6	0.12 ± 0.04	0.14 ± 0.04	0.123
20:3n-6 (DGLA)	1.20 ± 0.40	0.87 ± 0.32	<0.001
20:4n-6 (ARA)	6.33 ± 1.39	5.15 ± 1.07	<0.001
22:4n-6 (AdA)	0.16 ± 0.04	0.13 ± 0.04	<0.001
18:3n-3 (ALA)	0.29 ± 0.13	0.24 ± 0.16	0.094
20:4n-3 (ETA)	0.078 ± 0.023	0.061 ± 0.030	<0.001
20:5n-3 (EPA)	0.74 ± 0.25	0.73 ± 0.43	0.916
22:5n-3 (DPA)	0.44 ± 0.10	0.38 ± 0.079	0.007
22:6n-3 (DHA)	1.51 ± 0.51	1.24 ± 0.45	0.001

Pre KTx - just before kidney transplant surgery, 3m post KTx - 3 months after kidney transplantation. AdA-adrenic acid, ARA-arachidonic acid, BCFA-branched chain fatty acids, DGLA-dihomo-linolenic acid, DHA-docosahexaenoic acid, DI-desaturation index (18:1/18:0), DPA-docosapentaenoic acid, ECFA-even chain fatty acids, EPA-eicosapentaenoic acid, ETA-eicosatetraenoic acid, MUFA-monounsaturated fatty acids, OCFA-odd chain fatty acids, PUFA-polyunsaturated fatty acids, SFA-saturated fatty acids.
